# Retraction Note: A combined microfluidic deep learning approach for lung cancer cell high throughput screening toward automatic cancer screening applications

**DOI:** 10.1038/s41598-025-09817-y

**Published:** 2025-07-07

**Authors:** Hadi Hashemzadeh, Seyedehsamaneh Shojaeilangari, Abdollah Allahverdi, Mario Rothbauer, Peter Ertl, Hossein Naderi-Manesh

**Affiliations:** 1 Nanobiotechnology Department, Faculty of Biosciences, Tarbiat Modares University, Tehran, Iran; 2 Biomedical Engineering Group, Department of Electrical Engineering and Information Technology, Iranian Research Organization for Science and Technology (IROST), P.O. Box 33535111, Tehran, Iran; 3 Biophysics Department, Faculty of Biosciences, Tarbiat Modares University, Tehran, Iran; 4 Karl Chiari Lab for Orthopaedic Biology, Department of Orthopedics and Trauma Surgery, Medical University of Vienna, Währinger Gürtel 18-22, 1090 Vienna, Austria; 5 Institute of Applied Synthetic Chemistry and Institute of Chemical Technologies and Analytics, Faculty of Technical Chemistry, Vienna University of Technology, Getreidemarkt 9/163-164, 1060 Vienna, Austria

Retraction of: *Scientific Reports* 10.1038/s41598-021-89352-8, published online 07 May 2021

The Editors have retracted this Article.

Following publication, the Editors were alerted to the use of non-standard phrasing which deviates from established scientific terminology. Investigation by the Editors have uncovered further issues, as outlined below:

- Some text appears to be reproduced verbatim or inappropriately paraphrased from previously-published work with no overlapping authors such as^1,2^.
- Some microscopy images in Figure 2 do not show DAPI staining and therefore appear to be inconsistent with the staining protocol as described in the methods. Further clarifications provided by the Authors in this regard were also discrepant.
- Number of images in the augmented datasets used for training the models are not reported in the Article. The Editors were also unable to conclusively confirm the numbers as the Authors did not provide the augmented dataset or laboratory records as requested.

The Editors therefore no longer have confidence in the findings presented in the Article.

Peter Ertl and Hossein Naderi-Manesh did not respond to correspondence regarding this retraction. The other Authors disagree with this retraction.
